# Fecal Microbiome and Metabolomic Profiles of Mixed-Fed Infants Are More Similar to Formula-Fed than Breastfed Infants

**DOI:** 10.3390/microorganisms13010166

**Published:** 2025-01-14

**Authors:** Mei Wang, Negin Valizadegan, Christopher J. Fields, Sharon M. Donovan

**Affiliations:** 1 Department of Food Science & Human Nutrition, University of Illinois, Urbana, IL 61801, USA; meiwang@illinois.edu; 2 High-Performance Biological Computing, University of Illinois, Urbana, IL 61801, USA; valizad2@illinois.edu (N.V.); cjfields@illinois.edu (C.J.F.); 3 Division of Nutritional Sciences, University of Illinois, Urbana, IL 61801, USA

**Keywords:** delivery mode, infant feeding, breastfeeding, gut microbiome, gut metabolome, metagenomic sequencing

## Abstract

Many infants consume both human milk and infant formula (mixed-fed); however, few studies have investigated how mixed feeding affects the gut microbiome composition and metabolic profiles compared to exclusive breastfeeding or formula feeding. Herein, how delivery mode and early nutrition affect the microbiome and metabolome of 6-week-old infants in the STRONG Kids2 cohort was investigated. Fecal samples were collected from exclusively breastfed (BF; n = 25), formula-fed (FF; n = 25) or mixed-fed (MF; n = 25) participants. Within each feeding group, infants were either delivered vaginally (VD; n = 13) or by Cesarean section (CS; n = 12). Feeding mode affects the fecal microbiome diversity, composition, and functional potential, as well as metabolomic profiles regardless of delivery mode. Alpha and beta diversity of MF differed from that of BF (*p* < 0.05) but were comparable to FF infants. Functional analyses have shown 117 potential metabolic pathways differed between BF and FF, 112 between BF and MF, and 8 between MF and FF infants (*p* < 0.05, *q* < 0.10). Fecal metabolomic profiles of MF and FF clustered together and separated from BF infants. In total, 543 metabolites differed between BF and FF, 517 between BF and MF, and 3 between MF and FF (*p* < 0.05, *q* < 0.10). Delivery mode affected overall microbial composition (*p* = 0.022) at the genus level and 24 potential functional pathways, with 16 pathways being higher in VD than CS infants (*p* < 0.05, *q* < 0.10). Metabolomic analysis identified 47 differential metabolites between CS and VD, with 39 being lower in CS than VD (*p* < 0.05, *q* < 0.10). In summary, fecal microbiota composition and function and metabolite profiles of 6-week-old MF infants are closer to FF than BF infants.

## 1. Introduction

Human milk is the preferred form of nutrition for infants. The 2020–2025 Dietary Guidelines for Americans and the American Academy of Pediatrics (AAP) recommend exclusive breastfeeding infants for about the first 6 months, with continued breastfeeding along with the introduction of appropriate complementary food for at least 1 [1] or 2 years [2]. However, many infants do not meet these recommendations. Among infants born in 2019, breastfeeding initiation was 62.6%. By 6 months of age, only 24.9% of infants were exclusively breastfed and most infants were either exclusively formula-fed (41.2%) or mixed-fed (33.9%) [3]. Both human milk and infant formula provide essential nutrients to support the rapid growth and development of infants. However, infant formula lacks the bioactive components present in human milk, such as human milk oligosaccharides (HMOs), immune proteins, cytokines, and milk microbiota, which protect infants against pathogenic infections, allergic, and autoimmune diseases in childhood, and shape the composition and function of the infant gut microbiota [4,5].

The assembly of the human gut microbiota is a dynamic process, and the primary changes occur during the first 2- to 3- years-of-life [6,7,8]. The composition and activity of infant gut microbiota play a critical role in infant gastrointestinal and cognitive development, maturation of the immune system, and metabolic programming [9]. Dysbiosis of the gut microbiota in early life is associated with a wide spectrum of conditions in childhood and later in life, such as necrotizing enterocolitis (NEC) in preterm infants, inflammatory bowel diseases (IBDs), eczema, asthma, diabetes, obesity, and neurological disorders [10,11,12]. Colonization by the gut microbiota in early life is influenced by perinatal, peripartum, and postnatal factors, including maternal diet and weight gain during pregnancy, gestation age, delivery mode, feeding type, the introduction of solid, antibiotic exposure, and use of pre-and probiotics [10,13,14,15,16]. Feeding type is one of the major determinants of early infant gut microbiota, and the influence of breastfeeding and formula feeding on microbiota composition and functional potential is well documented [9,15]. Yet, fewer studies have investigated the effect of mixed feeding on microbiota composition and the results from these studies are inconsistent [9,17]. Moreover, less attention has been paid to the role of feeding mode on the fecal metabolic profiles of infants [18,19].

Cesarean section (CS) birth rates continue to rise globally, accounting for 21% of all childbirth [20]. In the US, the CS rate was 32.1%, well surpassing the WHO’s ideal acceptable rate of 10–15% [21,22]. CS reduces both maternal and neonatal morbidity and mortality when medically indicated; however, CS rates above recommendations are associated with a higher frequency of immune and metabolic conditions, such as asthma, food allergy, type 1 and type 2 diabetes and obesity, all of which are related to early life microbial dysbiosis [23,24,25]. It is hypothesized that abnormal microbiota colonization in CS infants alters the host metabolism and natural immune development, leading to increased susceptibility to metabolic and immune disorders [23,24]. Numerous studies have shown that delivery mode shapes the initial gut microbial colonization [26,27,28]. During the first hours and days postpartum, the microbiota of vaginal delivery (VD) infants typically resembles that of their mother’s vaginal and fecal microbiota [26,29,30]. In contrast, the microbiota of neonates born by CS is more similar to that found on maternal skin and the hospital environment [26]. The impact of delivery mode on the gut microbiota beyond the first days of life has been extensively studied but the results from those studies remain inconsistent [24,27,31,32]. Furthermore, most of the studies have focused on the microbiota composition using 16S rRNA gene sequencing, and data on the impact of delivery mode on the metabolic activity of gut microbiome in early life are limited [19,27,33].

The fecal metabolome provides a functional readout of microbial activity and reflects metabolic interactions between the host, diet, and gut microbiota [34]. This study aimed to investigate how delivery mode and early life nutrition shape the infant fecal microbiome and metabolome using shotgun metagenomic sequencing and untargeted metabolomics. We hypothesized that both delivery mode and feeding type would affect fecal microbiota composition, function potential, and metabolic profile. We further hypothesized that fecal microbiome and metabolomic profiles of mixed-fed (MF) infants would be intermediate between exclusively formula-fed (FF) and exclusively breastfed (BF) infants.

## 2. Materials and Methods

### 2.1. Study Participants

Study participants were drawn from The Synergistic Theory Research Obesity and Nutrition Group (STRONG) Kids 2 birth cohort study, which aims to examine multilevel predictors of weight trajectories and dietary habits over the first seven years of life (n = 468) [35]. The study is registered as a clinical trial with the U.S. National Library of Medicine registry under the number NCT03341858. For this study, 75 healthy term infants who were either exclusively breastfed (BF; n = 25), exclusively formula-fed (FF; n = 25), or mixed-fed (receiving both human milk and infant formula from birth to the time of sample collection) (MF; n = 25) were enrolled. Infants within each feeding group were either delivered vaginally (VD; n = 13) or by Cesarean section (CS; n = 12). Attempts were made to reduce the introduction of selection bias relative to the full cohort, except that the subsample for this study was overrepresented for MF and FF infants and infants born by CS, by design. In the full STRONG Kids 2 cohort, 67.7%, 13.9%, and 17.9% of infants were exclusively BF, FF, or MF at 6 wk, respectively, compared with 33.3% in each group in this study. Additionally, 48% of the infants were delivered by CS in the current study compared to 25.6% in the full cohort [35].

### 2.2. Questionnaire Data

An online questionnaire completed by the parent was used to collect self-reported demographic characteristics, including maternal age, prepregnancy height and weight, infant sex assigned at birth, gestational age at delivery, race and ethnicity, and infant weight and length at birth, antibiotic usage, delivery mode, and feeding practices at birth, 1 week and 6 weeks of age. The amount and proportion of human milk that MF infants consumed were estimated based on the response to the question: in the past 7 days, how often was your baby fed human milk or formula? Data were recorded as numbers of feedings per day. The research staff recorded infant weight and length during a home visit at 6 weeks of age. Maternal body mass index was calculated. Infant age and sex weight-for-length z-scores (WFLz) were derived according to the WHO growth standard for children using the childsds (v0.8.0) package in R [36].

### 2.3. Sample Collection

At 6 weeks postpartum, a freshly voided stool sample was collected from the diaper in the participant’s home as previously described [35]. A sample collection kit, which included a disposable collection pad, nitrile powder-free gloves, screw-top tubes, a sampling spoon, and a storage box, was provided by the research staff, and mothers were instructed to wear gloves and use the sterile spoons to transfer the stool sample from the diaper into the tubes. Tubes were then placed into the storage box, which was placed into a sealable bag and stored in the participant’s home freezer (−20 °C). Samples were picked up within 2 to 24 h of collection and stored at −80 °C until further processing. Participants were living in East Central Illinois and samples were collected between 2013 and 2017.

### 2.4. Shotgun Metagenomic Sequencing and Analysis

#### 2.4.1. DNA Extraction

DNA was extracted from ~200 mg of feces by the QIAamp Fast DNA Stool Mini Kit (Qiagen, Valencia, CA, USA) in combination with bead beating on the FastPrep-24 System (MP Biomedicals, Carlsbad, CA, USA) as previously described [37]. DNA concentration was measured with a Qubit 3.0 Fluorometer (Thermo Fisher Scientific, Waltham, MA, USA) and DNA quality was checked on a 1% agarose gel in TBE buffer.

#### 2.4.2. Construction of Shotgun Genomic Libraries and Sequencing

Library contraction and sequencing were carried out at the DNA Services Lab at the University of Illinois Urbana-Champaign. The shotgun genomic libraries were constructed from 200 ng of DNA using unique dual-indexed adaptors as previously described [38]. After genomic DNA was sonicated with a Covaris ME220 (Covaris LLC, Woburn, MA, USA) to an average fragment size of 300 bp, the DNA library was constructed from fragmented DNA with KAPA Hyper Library Preparation Kit (Roche Molecular Systems, Pleasanton, CA, USA). During the library construction, the unique dual-indexed adaptors from Illumina (San Diego, CA, USA) were used to avoid index switching. The individually barcoded libraries were amplified with 5 cycles of PCR and run on a Fragment Analyzer (Agilent Technologies, Santa Clara, CA, USA) to confirm the absence of free primers and primer dimers and the presence of DNA of the expected size range. Libraries were pooled in equimolar concentration, size selected on a 2% agarose gel (220 bp to 280 bp in length) and quantitated by qPCR as described by Ellis et al. [38]. The pooled barcoded libraries were loaded on a lane of NovaSeq 6000 system (Illumina, San Diego, CA, USA) for cluster formation and sequencing at a length of 150 nt from each side of the DNA fragments. The fastq read files were generated and demultiplexed with the bcl2fastq v2.20 Conversion Software (Illumina, San Diego, CA, USA).

#### 2.4.3. Sequence Processing and Bioinformatics

Adapter sequences and low-quality reads were trimmed on both reads with Trimmomatic [39]. Quality control was performed before and after the trimming steps with FastQC and MultiQC with default parameters [40,41]. Trimmed paired reads were merged with vsearch (https://github.com/torognes/vsearch (accessed on 25 June 2020) [42]), and sequences generated from the human genome were removed using KneadData (version 6.1; https://github.com/biobakery/kneaddata (accessed on 12 July 2020)). Taxonomic classification and functional profiling of metagenomic samples were performed using MetaPhlAn2 (https://github.com/biobakery/MetaPhlAn2 (accessed on 11 October 2020) [43]) and HUMAnN2 (https://huttenhower.sph.harvard.edu/humann2 (accessed on 15 November 2020) [44]), respectively, using the default settings. HUMAnN2 reported the abundances of gene families from the UniRef90 protein database and constructed pathways based on the gene family mapping to the MetaCyc metabolic pathway database (version 24.1) [45]. Taxonomic and pathway abundances of each sample were merged and normalized to relative abundance. Each pathway was categorized based on its parent class in the MetaCyc database and assigned to a superclass and subclass category.

### 2.5. Fecal Metabolomics Analysis

#### 2.5.1. Sample Preparation and UPLC-MS/MS Analysis

Infant fecal samples (100 mg) were submitted to Metabolon Inc. (Durham, NC, USA) for global metabolite profiling as described previously [34,46]. Samples were prepared using the automated MicroLab STAR^®^ system (Hamilton, Reno, NV, USA). Briefly, recovery standards were added prior to the first step in the extraction process for quality control (QC) purposes. Proteins were precipitated with methanol under vigorous shaking for 2 min followed by centrifugation to remove protein, dissociate small molecules bound to protein or trapped in the precipitated protein matrix, and recover chemically diverse metabolites. The resulting extract was divided into five fractions, one for each individual ultra-high liquid-phase chromatography coupled with tandem mass spectrometry (UPLC-MS/MS) analysis as described below and one for backup. The fractions were briefly evaporated to remove the organic solvent and stored overnight under nitrogen before preparation for analysis.

The following controls were analyzed in concert with the experimental samples: (1) a pooled sample generated by taking a small volume of each experimental sample to serve as technical replicate; (2) extracted water samples served as process blanks; (3) a cocktail of quality control (QC) standards that were spiked into every analyzed sample, allowed instrument performance monitoring, and aided chromatographic alignment. Instrument variability was determined by calculating the median relative standard deviation (RSD) for the standards added to each sample before injection into the mass spectrometers.

All methods utilized a Waters ACQUITY ultra-performance liquid chromatography and a Thermo Scientific Q-Exactive high resolution/accurate mass spectrometer interfaced with a heated electrospray ionization (HESI-II) source and Orbitrap mass analyzer operated at 35,000 mass resolution (Thermo Fischer Scientific, Waltham, MA, USA) as described by Arrieta et al. [46]. On the day of analysis, the sample extract was dried and then reconstituted in solvents compatible with each of the four methods. Each reconstitution solvent contained a series of standards at fixed concentrations to monitor injection and chromatographic consistency. The aliquots were analyzed using (1) acid-positive ion conditions optimized for more hydrophilic compounds; (2) acid-positive ion conditions optimized for more hydrophobic compounds; (3) basic negative-ion-optimized conditions using a separate dedicated C18 column basic negative ion mode ESI; (4) negative ionization following elution from a HILIC column. The MS analysis alternated between MS and data-dependent MSn scans using dynamic exclusion. The scan range varied slightly between methods but covered 70–1000 *m*/*z* [46].

#### 2.5.2. Data Extraction, Compound Identification, and Quantification

Raw data were extracted, peak-identified and QC processed using Metabolon’s hardware and software. Compounds were identified by automated comparison of the ion features of samples to library entries of purified standards that contained retention time/index, mass-to-charge ratio (*m*/*z*), and chromatographic data (including MS/MS spectral data) using software developed at Metabolon. Biochemical identifications were based on three criteria: retention index within a narrow RI window of the proposed identification, accurate mass match to the library ±10 ppm, and the MS/MS forward and reverse scores between the experimental data and authentic standards. The MS/MS scores were based on a comparison of the ions present in the experimental spectrum to the ions present in the library spectrum. Peaks were quantified using the area under the curve.

Knowledge-based pathway annotations from the metabolomics platform (Metabolon Inc.) were used for each metabolite. Each known metabolite was annotated with one of 73 sub-pathways, which represent metabolic pathways or biochemical subclasses of the compounds. In addition, each sub-pathway was assigned to one of eight super-pathways: ‘Amino acid’, ‘Lipid’, ‘Carbohydrate’, ‘Nucleotide’, ‘Peptide’, ‘Energy’, ‘Cofactors and vitamins’, and ‘Xenobiotics’. These pathway annotations have frequently been used in previous studies investigating data from the same platform [47].

### 2.6. Statistical Analysis

Demographic data, including gestational age at birth, WFLz, maternal age and prepregnancy BMI, and alpha diversity indices were analyzed using the PROC MIXED procedure of SAS (version 9.4; SAS Institute). Alpha diversity (Shannon, Inverse Simpson and evenness indices) was calculated using the vegan R package (version 2.6-4) [48]. The statistical model included feeding, delivery and the interaction between feeding and delivery. When feeding was significant, the post hoc Tukey test was used to determine differences among feeding groups. As inverse Simpson indices were not normally distributed, log_10_ transformation was applied. Differences in infant sex, ethnicity and antibiotic intake between feeding type or delivery mode were analyzed by Chi-squared test using the chisq.test function of the R package of stats. Data are presented as means ± SEMs unless indicated otherwise. A *p*-value of ≤0.05 was considered statistically significant.

The effect of feeding and delivery modes on beta diversity was evaluated with principal coordinate analysis (PCoA) and permutational multivariate analysis of variance (PERMANOVA) using Bray–Curtis dissimilarity matrices. Bray–Curtis dissimilarity was calculated from the relative abundances of bacterial genera or species using the R package of phyloseq [49]. PCoA plots were generated using the phyloseq function of ordinate. PERMANOVA were performed in QIIME2 [50] using the qiime diversity plugin with the adonis method. Pairwise comparisons across different feeding types were conducted with qiime diversity beta-group significance. Statistical significance was set at *p* ≤ 0.05.

Multivariate analysis by linear models (MaAsLin 2.0) [51] was used to determine the associations between delivery mode and feeding type and the relative abundance of microbial taxa or metabolic pathways. The relative abundance of data was arcsine-square root transformed and the taxa or pathways presented in <10% of samples were excluded from this analysis. All *p*-values were corrected for multiple testing by the Benjamini–Hochberg false discovery rate (FDR), and *p* < 0.05 and *q* (adjusted *p*) < 0.10 were considered statistically significant.

Metabolite profiles in infant feces were quantified in terms of relative abundance and rescaled to set the median equal to 1. The missing values were imputed with the minimum observed value for each compound. Data were log-transformed prior to statistical analysis. Principal component analysis (PCA) was used to discriminate samples from different feeding groups (BF, MF, FF) or delivery modes (VD, CS). Random forest (RF) was applied to estimate how well one can predict sample classes in a new data set (prediction accuracy). Two-way ANOVA and post hoc ANOVA contrasts were performed to identify metabolites that differed significantly between experimental groups. An estimate of FDR (*q*-value) was calculated to account for the effect of multiple comparisons. Data were expressed as log_2_-fold change and metabolites with *p* < 0.05 and *q* < 0.10 were considered statistically significant. Two-way ANOVA was performed in Array Studio (Omicsoft, Cary, NC, USA). PCA and random forest were performed using R packages of stats and randomForest, respectively. MetOrigin online platform [52] was used to discriminate the origin of metabolites and perform origin-based metabolic pathway enrichment analysis (MPEA). Pathways with *p* ≤ 0.05 were reported as statistically significant in MPEA analysis. MetaOrigin integrates seven metabolite databases, including Kyoto Encyclopedia of Genes and Genomes (KEGG), the human metabolome database (HMDB), the Biochemically, Genetically and Genomically structured knowledgebase (BiGG), Chemical Entities of Biological Interest (ChEBI), the Food Database (FoodDB), Drugbank, and the Toxin and Toxin Target Database (T3DB). For Simple MetaOrigin Analysis (SMOA), a list of metabolites with their KEGG or HMDB IDs was used as input.

## 3. Results

### 3.1. Demographics

Participant demographics are shown in Table 1. Most (65.3%) of the infants were non-Hispanic/Latino white and 48% were male. There was no significant difference in gestation age at birth, antibiotic intake, WFLz at birth and 6 weeks of age among feeding groups or between VD and CS infants (*p* > 0.05). Mothers of BF infants were older than FF, with MF being intermediate (*p* < 0.05). The prepregnancy BMI of CS mothers was higher than that of VD mothers (*p* = 0.022). MF infants consumed 52.3 ± 6.5% of their intake as breastmilk (range 8.2–93.3%).

### 3.2. Feeding Practice and Delivery Mode Affected Infant Fecal Microbiome

#### 3.2.1. General Features of Metagenome

NovaSeq sequencing generated 2.8 billion paired-end reads from a total of 75 samples (range: 17.3–58.2 million per sample). After quality control filtering, merging of paired-end reads and removing of human sequences, 92.5% of reads (2.6 billion) were utilized for taxonomic classification and functional annotation using MetaPhlAn2 and HUMAnN2.

#### 3.2.2. Taxonomic Analysis

**Figure 1:**
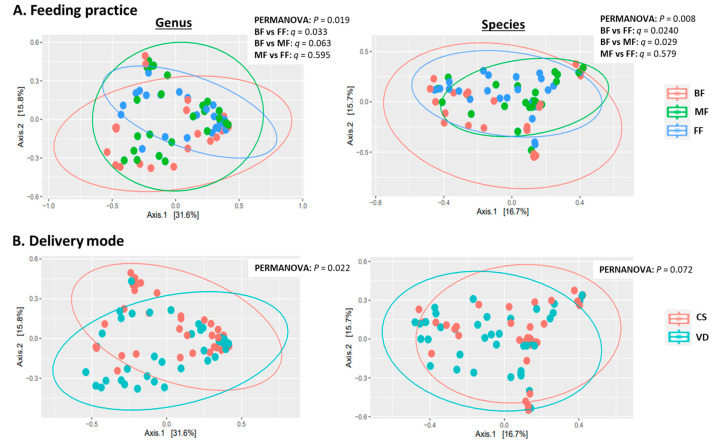
Effect of feeding practice (**A**) and delivery mode (**B**) on genus (**left**) and species (**right**) beta diversity in fecal samples of infants at 6 weeks of age. BF, breastfed; CS, cesarean section; FF, formula-fed; MF, mixed-fed; VD, vaginally delivered.

MetaPhlAn2 classified sequences into 111 microbial genera and 317 species. Statistical analyses indicated feeding practice and delivery mode independently influenced microbial alpha and beta diversity with no interaction (Table 2, Figure 1A). At both genus and species levels, alpha-diversity measures (Shannon, inverse Simpson and evenness indices) were lower in BF than in MF or FF infants (*p* < 0.05), while alpha diversity was similar between MF and FF *(p* > 0.73; Table 2). PCoA of Bray–Curtis dissimilarity generated from relative abundances of microbial genera and species are shown in Figure 1A. PERMANOVA revealed that feeding mode affected the overall microbial community structures (beta diversity) at both species and genus levels (*p* = 0.019 and *p* = 0.008, respectively). At the species level, the microbial composition of BF infants differed from those of FF and MF infants (BF vs. FF: *p* = 0.024; BF vs. MF: *p* = 0.029). Similar results were observed at the genus level with BF differing from FF (*p* = 0.033) and BF showing a tendency to differ from MF (*p* = 0.063). Overall microbial community structures were similar between FF and MF infants at both genus and species levels (*p* = 0.595 and *p* = 0.579, respectively; Figure 1A).

**Figure 2:**
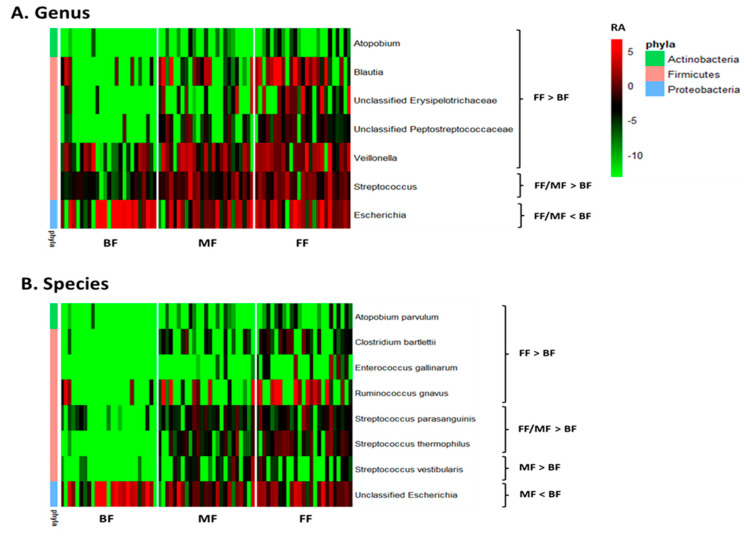
Effect of feeding practice on the relative abundances of microbial genera (**A**) and species (**B**) in fecal samples of infants at 6 weeks of age. BF, breastfed; FF, formula-fed; MF, mixed-fed; RA, log_2_ relative abundance.

Multivariate analysis by linear models indicated feeding practice influenced relative abundances of some microbial genera and species. Pairwise comparisons between feeding groups identified seven bacterial genera that differed between BF and FF and two between BF and MF. *Atopobium*, *Blautia*, *Veillonella*, *Streptococcus*, and unclassified Erysipelotrichaceae and Peptostreptococcaceae were lower, while *Escherichia* was higher in BF than FF infants (*p* < 0.05, *q* < 0.10; Figure 2A and Table S1). The differentiating genera between BF and MF were *Streptococcus* and *Escherichia* with *Streptococcus* being lower and *Escherichia* higher in BF (*p* < 0.05, *q* < 0.10; Figure 2A and Table S1). Six species, including *Atopobium parvulum* (renamed as *Lancefieldella parvula*), *Clostridium bartlettii* (*Intestinibacter bartlettii*), *Enterococcus gallinarum*, *Ruminococcus gnavus (Mediterraneibacter gnavus*), *Streptococcus parasanguinis* and *Streptococcus thermophilus* distinguished BF from FF, with their relative abundances being lower in BF than FF (*p* < 0.05, *q* < 0.10; Figure 2B and Table S2). Four species differentiated BF from MF with *Streptococcus parasanguinis*, *Streptococcus thermophilus* and *Streptococcus vestibularis* being lower and unclassified *Escherichia* being higher in BF than MF (*p* < 0.05, *q* < 0.10, Figure 2B and Table S2).

**Figure 3:**
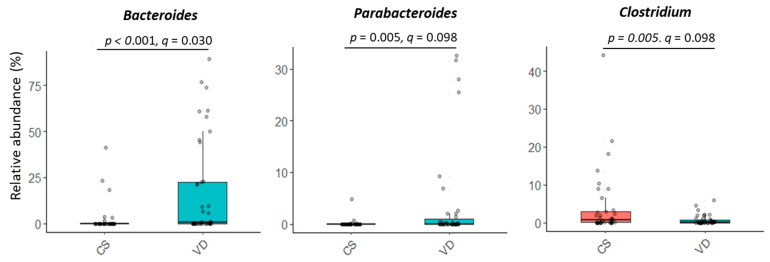
Effect of delivery mode on the relative abundances of microbial genera in fecal samples of infants at 6 weeks of age. At the time of publication, NCBI reclassified *Bacteroides* to include species of *Bacteriodes*/*Phocaeicola* and *Clostridium* was reclassified to include species of *Clostridium*/*Enterocloster*/*Hungatella* (Table S1; https://www.ncbi.nlm.nih.gov/Taxonomy/Browser/wwwtax.cgi (accessed on 27 December 2024)). CS, cesarean section; VD, vaginally delivered.

Delivery mode impacted overall microbial communities at genus level (*p* = 0.022; Figure 1B). A similar trend was observed at species level (*p* = 0.072, Figure 1B). Relative abundances of *Bacteroides* and *Parabacteroides* were higher, while *Clostridium* were lower in VD compared to CS infants (*p* < 0.05, *q* < 0.10; Figure 3 and Table S1). Delivery mode had no effect on alpha diversity (*p* > 0.41, Table 2).

#### 3.2.3. Functional Analysis

A total of 437 potential metabolic pathways were detected in the fecal metagenomes of infants by HUMAnN2. After removing pathways presented in <10% of the samples, 360 pathways were analyzed by MaAsLin2. Feeding practice affected relative abundances of 155 pathways. Among these pathways, 117 pathways were statistically different between BF and FF, 112 between BF and MF, and 8 between MF and FF infants (*p* < 0.05, *q* < 0.10; Table 3).

**Figure 4:**
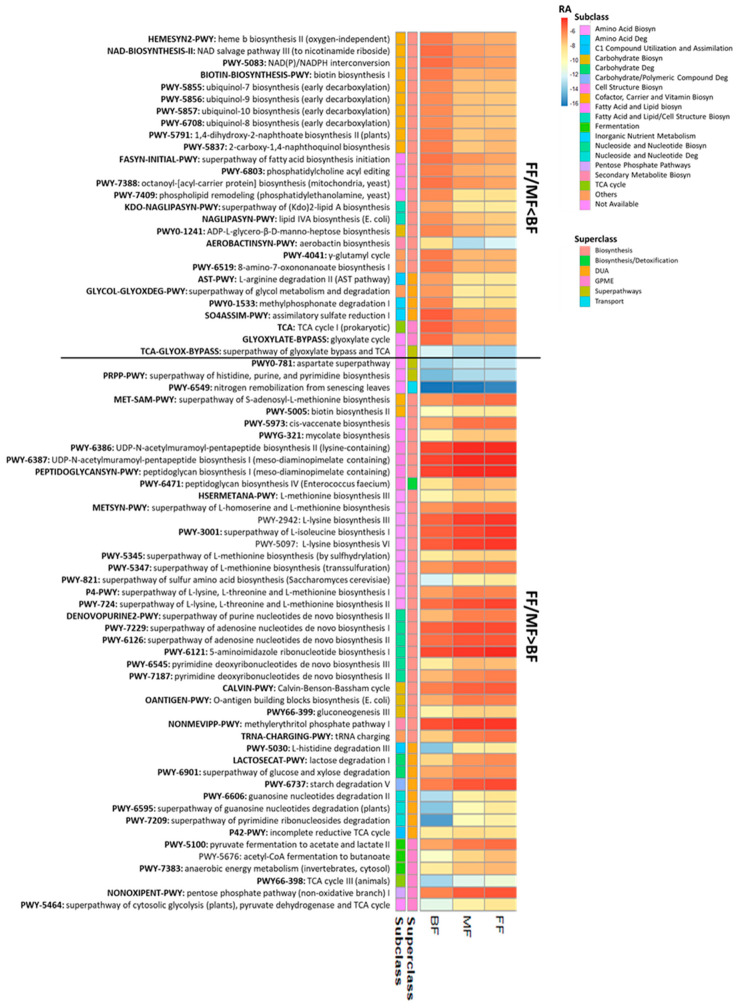
Fecal metabolic pathways differed between BF infants and infants fed only (FF) or some (MF) infant formula at 6 weeks of age (*p* < 0.05, *q* < 0.10). Biosyn, biosynthesis; BF, breastfed; Deg, degradation; DUA, degradation/utilization/assimilation; FF, formula-fed; GPME, generation of precursor metabolites and energy; MF, mixed-fed; RA, log_2_ mean relative abundance.

The relative abundances of 73 pathways differed between BF and FF/MF infants (*p* < 0.05, *q* < 0.10; Figure 4). Most pathways (n = 49; 67.1%) belonged to the superclass of biosynthesis, followed by degradation/utilization/assimilation (DUA), and generation of precursor metabolites and energy (GPME; Figure 4). Amino acid biosynthesis (n = 10), fatty acid and lipid (n = 8), nucleoside and nucleotide (n = 6), cofactor, carrier and vitamin (n = 12) were the main pathways that distinguished BF from FF/BF. Overall, the fecal microbiome of BF infants had lower relative abundances of pathways for biosynthesis of amino acids (such as lysine, threonine, methionine), nucleosides, and nucleotides, while FF/MF infants had lower proportions of pathways related to cofactors, carriers, and vitamin biosynthesis, particularly ubiquinol (Figure 4).

A total of 36 pathways differed between FF and BF with MF being the intermediate. Most of these pathways (n = 32; 88.9%) were lower in BF than FF infants (*p* < 0.05, *q* < 0.10; Figure S1). Compared to FF, BF infants had lower proportions of pathways associated with biosynthesis of amino acids, including L-glutamate and L-glutamine, and aromatic amino acids (n = 3), secondary metabolites (n = 4), nucleosides and nucleotides (n = 3), degradation of carbohydrates (n = 3) and fermentation (n = 4) (Figure S1). In contrast, 38 pathways significantly differed only between MF and BF with 31 (81.6%) being higher in BF infants (*p* < 0.05, *q* < 0.10; Figure S2). Pathways related to biosynthesis of amino acids (n = 3), carbohydrates (n = 2), cofactors, carriers, and vitamins (n = 9), especially menaquinol, and carbohydrate degradation(n = 3) were higher in BF than MF, but not FF (Figure S2).

Of the 360 pathways analyzed, only 8 pathways differed significantly between FF and MF/BF with all being higher in FF than MF or BF (*p* < 0.05, *q* < 0.10) (Table 3). Among these, 5 pathways (PWY-7200: superpathway of pyrimidine deoxyribonucleoside salvage, PWY-922: mevalonate pathway I, P125-PWY: superpathway of (R,R)-butanediol biosynthesis, PWY-6396: superpathway of 2,3-butanediol biosynthesis and PWY-2941: L-lysine biosynthesis II) were related to biosynthesis, one (P164-PWY: purine nucleobases degradation I) to DUA/GPME, one (PWY-7115: C4 photosynthetic carbon assimilation cycle, NAD-ME type) to GPME and one (ARGORNPROST-PWY: arginine, ornithine and proline interconversion) to interconversion.

**Figure 5:**
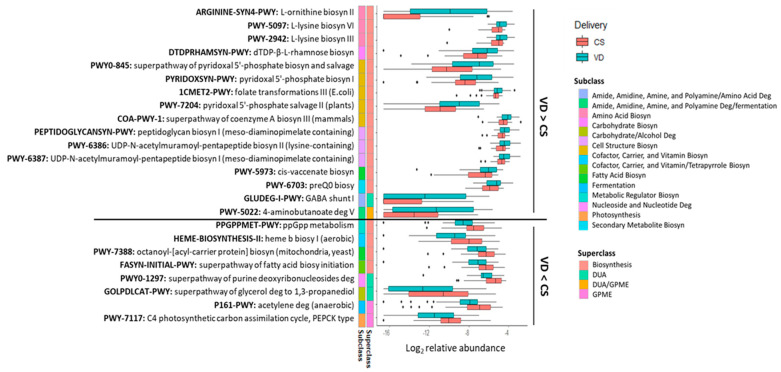
Fecal metabolic pathways differed between CS and VD infants at 6 weeks of age (*p* < 0.05, *q* < 0.10). Biosyn, biosynthesis; CS, cesarean section; Deg, degradation; DUA, degradation/utilization/assimilation; GPME, generation of precursor metabolites and energy; VD, vaginally delivered.

Delivery mode impacted 24 potential microbial metabolic pathways (*p* < 0.05, *q* < 0.10; Figure 5) with the relative abundances of 16 pathways being higher in the fecal microbiome of VD than CS infants (Figure 5). Most of the pathways (14 out of 16) were of the biosynthesis superclass. Of these 14 pathways, 3 belonged to amino acid biosynthesis (ARGININE-SYN4-PWY, PWY-2942, PWY-5097), 5 to the cofactor, carrier, and vitamin biosynthesis (PYRIDOXSYN-PWY, PWY0-845, 1CMET2-PWY, PWY-7204, COA-PWY-1), and 3 to the cell structure biosynthesis (PEPTIDOGLYCANSYN-PWY, PWY-6386, PWY-6387). The remaining three pathways were related to carbohydrate (DTDPRHAMSYN-PWY), fatty acid (PWY-5973) and secondary metabolite biosynthesis (PWY-6703). Two pathways belonged to the superclass of DUA and/or GPME (GLUDEG-I-PWY, PWY-5022). Eight pathways were lower in the fecal microbiome of VD than CS infants (*p* < 0.05, *q* < 0.10; Figure 5). From these, four were associated with the superclass of biosynthesis, including one related to the cofactor, carrier, and vitamin biosynthesis (HEME-BIOSYNTHESIS-II), two to the fatty acid biosynthesis (PWY-7388, FASYN-INITIAL-PWY) and one to the metabolic regulator biosynthesis (PPGPPMET-PWY). Two pathways belonged to subclass DUA, one associated with nucleoside and nucleotide degradation (PWY0-1297) and another with carbohydrate/alcohol degradation. Among the two pathways of GPME, one belonged to fermentation (P161-PWY) and the other to photosynthesis (PWY-7117).

### 3.3. Feeding Practice and Delivery Mode Influenced Infant Fecal Metabolites

Global metabolic profiling detected a total of 1000 metabolites with 804 compounds of known identity. Analysis of 1000 compounds by two-way ANOVA showed significant main effects of feeding (577 compounds) and delivery mode (47 compounds) (*p* < 0.05, *q* < 0.10), with no interaction between feeding and delivery (*q* > 0.1).

**Figure 6:**
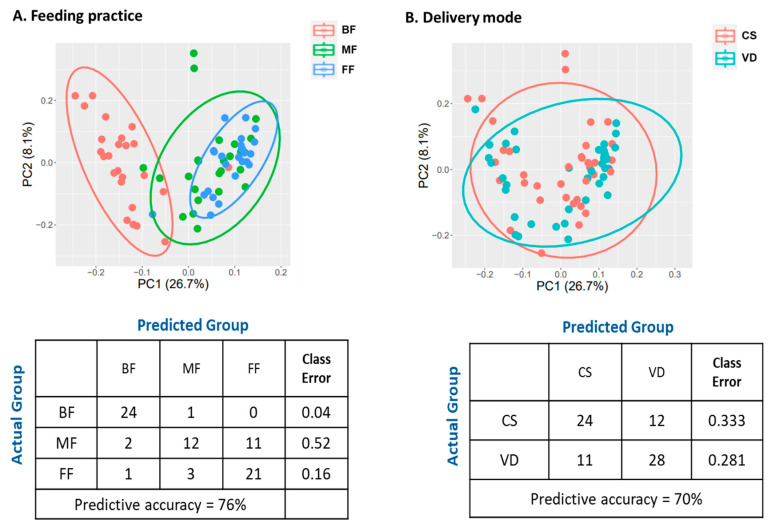
Effect of feeding practice (**A**) and delivery mode (**B**) on fecal metabolomic profiles of infants at 6 weeks of age. Plot of principle component analysis (**Top**), and random forest confusion matrix (**Bottom**). BF, breastfed; CS, cesarean section; FF, formula-fed; MF, mixed-fed; VD, vaginally delivered.

PCA revealed the metabolic profiles of FF and MF were clustered together, but clearly separated from BF infants (Figure 6A). These results were further supported by RF classification (Figure 6A). RF could accurately predict BF samples (24/25, class error rate 0.04) and FF samples (21/25, error rate 0.16). For MF samples, RF predicted 12 (48%) samples to MF, 11 (44%) to FF, and 1 (0.04%) to BF group with the class error rate of 0.52, suggesting MF samples were not distinguishable from FF samples.

ANOVA contrasts were applied to identify biochemicals that differed significantly between the feeding groups. Metabolites influenced by feeding practice were presented in Table 4 and Table S3). Compared to BF infants, FF and MF infants had a similar number of metabolites differing in abundance (543 and 517, respectively; *p* < 0.05, *q* < 0.10). Only three metabolites differed between FF and MF (*p* < 0.05, *q* < 0.10). The levels of 483 metabolites significantly differed between BF and FF/MF with 284 being higher in FF and MF than BF (*p* < 0.05, *q* < 0.10; Table S3). Chemicals belonging to super-pathways of lipids, amino acids, cofactors/vitamins, and carbohydrates were the main differentiating metabolites between the MF/FF and BF infants (Table 4).

The abundances of lactose, human milk oligosaccharides (HMOs) (Table 5), long-chain saturated and unsaturated fatty acids (Table 6), including docosahexaenoate, eicosapentaenoate and linolenate, and lysophospholipid were higher in BF compared to FF/MF (*p* < 0.05, *q* < 0.10; Table S3). In contrast, the levels of most amino acids including the metabolites derived from aromatic amino acids and lysine (Table 7), saturated fatty acids below C12 (Table 6), endocannabinoid, medium- and long-chain acyl carnitine, as well as the compounds associated with metabolisms of tocopherol, nicotinate and nicotinamide were lower in BF than MF/FF (*p* < 0.05, *q* < 0.10; Table S3).

A total of 60 chemicals differed only between BF and FF and 34 between BF and MF (*p* < 0.05, *q* < 0.10; Table S3). Among these, the abundances of nine secondary bile acids, including ursodeoxycholate (UDCA), isoursodeoxycholate (isoUDCA), ursocholate (UCA) and 7-ketodeoxycholate (7-ketoDCA), differentiated BF from FF (Table 8). These bile acids were higher in FF than BF, but not MF. The three metabolites distinguished MF from FF were docosadienoate, 2-hydroxypalmitate and 1,7-dimethylurate with the levels being higher in MF than FF infants (*p* < 0.05, *q* < 0.10; Table S3).

Simple MetOrigin analysis (SMOA) classified 804 metabolites with a known identity into six categories: host (n = 231), microbiota (n = 364), drug-related (n = 246), food-related (n = 636), environment (n = 60) and unknown (n = 150). Among the metabolites that belonged to the categories of host and microbiota, seven were specific to the host (human), 140 to the microbiota, and 244 were shared by both host and microbiota (co-metabolism). The original-based MPEA analysis indicated one human- (alpha-linolenic acid metabolism, *p* = 0.016) and five bacteria-specific metabolic pathways, including ubiquinone and another terpenoid-quinone biosynthesis, histidine metabolism, lysine degradation, pentose and glucuronate interconversions, as well as valine, leucine and isoleucine biosynthesis, were significantly different between BF and FF/MF (*p* < 0.05; Table 9). For the metabolites shared by both host and microbiota, pathway enrichment analysis identified 24 pathways that differed between BF and FF/MF (*p* ≤ 0.05; Table 9). Most of these pathways were associated with the metabolism of amino acids (proteinogenic amino acids, n = 7; other amino acids, n = 3), followed by lipid metabolism (n = 5). Four pathways that differed only between BF and FF were sulfur metabolism (*p* = 0.011), steroid biosynthesis (*p* = 0.014), citrate cycle (*p* = 0.02) and valine, leucine and isoleucine biosynthesis (*p* = 0.029). Pathways differentiating BF from MF, but not FF, were vitamin B6 and thiamine metabolism (*p* = 0.028 and *p* = 0.05, respectively) and monobactam biosynthesis (*p* = 0.05; Table 9). No pathway significantly differed between FF and MF (*p* > 0.05).

PCA of the metabolites of VD and CS infants revealed no distinct clustering by delivery mode (Figure 6B). Furthermore, RF did not accurately bin the CS and VD samples (class error rates: 0.333 and 0.281 for CS and VD, respectively; Figure 6B). ANOVA contrast identified 47 differential metabolites between CS and VD infants, with 39 being lower and eight being higher in CS than in VD (*p* < 0.05, *q* < 0.10; Table S4). Most of the significant compounds (86%) belonged to the super pathways of lipid, amino acid, carbohydrate, and nucleotide metabolism (Table 10). MPEA analysis identified three microbiota-specific (valine, leucine, and isoleucine biosynthesis, pantothenate and CoA biosynthesis, and pyruvate metabolism) and four co-metabolism pathways, including sphingolipid metabolism, pyrimidine metabolism, alanine, aspartate and glutamate metabolism and purine metabolism, were significantly associated with delivery mode (Table 11; *p* < 0.05).

## 4. Discussion and Conclusions

The establishment of infant gut microbiota is influenced by various factors, plays a vital role in maintaining host homeostasis, and has both short- and long-term health effects on the host. Shotgun metagenomic sequencing reveals microbial composition and functional potential. However, this approach does not measure the actual metabolic activity of the microbes [34]. Thus direct measurement of fecal metabolites can complement metagenomic sequencing and provide a functional readout of gut microbial metabolism [34]. However, the existing literature on how environmental factors, including delivery mode and early life nutrition, affect infant gut metabolic profiles is limited [15,28]. Using shotgun metagenomic sequencing and untargeted metabolomics, our study demonstrated that delivery mode and feeding type independently affected infants’ fecal microbiome and metabolome at 6 weeks of age.

As expected, we observed feeding mode strongly influenced alpha and beta diversity, taxonomic composition, and metabolic capacity of infant fecal microbiome. In our study, the alpha-diversity indices (Shannon, inverse Simpson, and evenness) of MF infants were comparable to FF but higher than BF infants. Additionally, PCoA and PERMANOVA revealed that the overall microbial structure of MF infants differed from that of BF infants but not FF infants. Pairwise comparisons between feeding groups identified seven bacterial genera and six species that differed between BF and FF, two genera and four species between BF and MF, and none between FF and MF. Furthermore, functional analyses uncovered 117 potential metabolic pathways that were statistically different between BF and FF, 112 between BF and MF, but only eight between MF and FF infants. Taken together, these results show the microbiome of MF infants was closer to that of FF than BF infants at 6 weeks of age.

Numerous studies have shown that infant microbiota composition and functional capacity differ between BF and FF infants [9]. However, relatively few studies have investigated how mixed feeding affects the gut microbiome composition, diversity, and metabolic function compared to exclusive breastfeeding or formula feeding. Using metagenomic sequencing, we observed that the alpha-diversity indices of MF or FF infants were higher than BF infants, which is in line with the result from a meta-analysis showing that non-exclusively breastfed infants are associated with increased alpha diversity [53]. In this study, we showed alpha diversity was similar between MF and FF infants, which contrasts the results from several other studies [8,54,55]. In the CHILD (the Canadian Healthy Infant Longitudinal Development) cohort (n = 996), fecal microbiota alpha-diversity measures (ACE, Chao1, Simpson, Shannon) of 3-mo-old MF infants were intermediate between BF and FF infants [54]. However, in the same CHILD cohort with a larger sample size (n = 3455), others reported that FF infants had higher alpha diversity (Shannon indices) compared with those who were MF or exclusively BF [55], which confirms the findings from the TEDDY (The Environmental Determinants of Diabetes in the Young) cohort study [8].

Consistent with the study of Madan et al. [56], MF and FF infants herein exhibited similar microbiota community structures at both the genus and species levels and were significantly different from the BF infants. This contrasts with some previous reports in which the overall microbiota structure of MF differed from both BF and FF infants [54,55] or was comparable to BF infants [57]. These contradictory observations may partly result from how MF was defined in different studies. In this study, MF infants were fed both human milk and infant formula from birth to the time of sample collection, while in other studies, the MF group included infants who received both human milk and formula at or prior to the time of stool collection [54,55,56,57]. Additionally, other factors, such as the number of infants studied, infant age, geographical location (USA, Canada, China), percentage of human milk that infants daily consumed, the human milk microbiota composition, and the analytic technology (metagenomics vs. 16S rRNA gene sequencing) may also influence the microbiota composition within MF infants.

The literature on how feeding mode, especially mixed feeding, affects the infant gut metabolome is very limited [19,57,58]. A recent study profiling the fecal metabolome of 121 US infants at 6 weeks of age reported that the stool metabolomic profile of BF infants was distinct from that of infants who were either FF or MF, but no significant difference between the metabolomic profiles of FF and MF infants [19], which agrees with our study. Using untargeted metabolomics analysis, we observed a clear separation of the global fecal metabolite profiles by feeding practice, as observed in the microbiome analysis. Both PCA and RF analyses indicated that fecal metabolite profiles of two groups receiving infant formula (FF and MF) were similar but significantly different from those of BF infants. Additionally, the numbers of differential metabolites were higher between BF and MF compared to between MF and FF (517 vs. 3), indicating that not only was the fecal microbiome composition and functional potential but also the actual metabolite activity, of MF was closer to FF than BF infants.

Differences in the microbiota composition between CS and VD infants were reported in many studies; however, the bacterial groups that were affected differed between studies. Our results confirmed that delivery mode is associated with infant fecal microbiome in early life. We observed overall microbial composition differed between VD and CS infants, which agrees with earlier studies [7,32,56,59,60]. Consistent with previous observations, we also found CD infants were depleted of the genera of *Bacteroides* and *Parabacteroides* [7,8,27,32,61]. *Bacteroides* is a predominant genus in the gut microbiota of human adults (mean relative abundance of 14.9%), while its abundance varies greatly in infant feces with some infants being dominated by *Bacteroides* [62,63]. *Bacteroides* play a critical role in the fermentation of non-digested carbohydrates, such as HMO, production of short-chain acids, and immune maturation [64,65,66]. Lower abundances of *Bacteroides* in infancy are linked to an increased risk of asthma and obesity later in life [56]. *Parabacteroides* is one of the 26 major genera detected in health individuals (≥3 years old) in 12 countries, with a mean abundance of 1.3% [62]. Species of *Parabacteroides* have been reported to be related to human health. For example, decreased levels of *Parabacteroides distasonis* in the human gut were associated with neonatal cholestasis disease, non-alcoholic fatty liver disease (NAFLD), inflammatory bowel disease (IBD), obesity and metabolic syndrome [67].

In this study, alpha-diversity measures were similar between VD and CS infants, which concurs with the studies of Reyman et al. [32] but contrasts with several earlier reports that CS infants exhibited decreased alpha diversity during early life [7,68]. Additionally, previous studies showed that, compared to VD infants, CS infants harbored a lower abundance of the *Bifidobacterium* during the first months of life [27,59,61,69]. However, we found *Bifidobacterium* was dominant in the microbiota of both VD and CS infants (mean relative abundance 36.4% and 35.3%, respectively) and the proportion of *Bifidobacterium* was similar between the two delivery modes, consistent with the previous results from infants of similar age [7,56,68]. Different confounding factors, such as feeding mode, infant age, geography, ethnicity, maternal antibiotic exposure, and experimental methods (DNA extraction methods, primers for 16S sequencing, sequencing technology), may partly explain the discrepancy in the results from different studies [24].

Few studies have investigated the relationship between delivery mode and infant fecal metabolome. A study of 6-week-old US infants (n = 121) observed differences in global metabolomic profiles between VD and CS infants. Comparisons between the two groups showed that 32 metabolites and four metabolic pathways distinguished VD from CS infants [19]. In another study, Li and colleagues examined the metabolites of 60 Chinese infants (n = 60) aged 2 to 26 weeks, reporting that 40 metabolites and 5 metabolic pathways were affected by delivery mode [33]. Consistent with the previous studies that used ANOVA contrasts and MetaOrigin analyses, we found that delivery mode impacted fecal metabolites and their representative metabolic pathways. Herein, 47 metabolites and seven metabolic pathways were identified to be associated with delivery mode. Previous studies have shown that pyruvate metabolism and glycolysis/gluconeogenesis pathways were enriched in VD compared to CS infants [19,33]. Our study observed the enrichment of pyruvate metabolism but not the glycolysis/gluconeogenesis pathway in VD infants. In contrast with the findings of Hoen et al. [19] that overall metabolomic profiles differed between VD and CS infants using PCA and random forest, we found that overall metabolite profiles were similar between the two delivery modes. These discrepancies among the studies could be due to differences in infant age, feeding mode, geographic location, technology used for metabolite profiling (NMR vs. LC-MS), and statistical methods applied for data analyses. More longitudinal cohort studies are needed to explore the effect of delivery mode on metabolic activity of gut microbiota in early life.

In cases where feeding practice affected individual fecal metabolites, we typically found the levels of fecal metabolites differed between BF and FF or BF and MF infants. One exception was the abundance of nine metabolites involved in secondary bile acid metabolism, including UDCA, isoUDCA, UCA, and 7-ketoDCA, which differed between BF and FF, but not between BF and MF infants. Compared to BF infants, these secondary bile acids were higher in FF, which supports previous reports of higher excretion of secondary bile acids in FF than in BF infants [70,71]. Primary bile acids are made from cholesterol in the liver and transformed into secondary bile acids by the gut microbiota [72]. Bile acids are important determinants of the community structure and function of gut microbiota, which in turn regulates the composition of the bile acid pool [73]. Bile acids are also essential for the digestion and absorption of lipids and fat-soluble vitamins and function as signaling molecules in various metabolic and inflammatory responses [72,74]. Early studies have demonstrated that secondary bile acids affect human health outcomes. A higher abundance of secondary bile acids is associated with colorectal cancer, while reduced levels of secondary bile acids in feces are linked to *Clostridium difficile* (*Clostridioides difficile*) infection, IBD, and metabolic syndrome in human adults [73,75]. Furthermore, the level of secondary bile acids was reported to be lower in the feces of children with NAFLD than those of healthy controls, suggesting a role of secondary bile acids in the development of pediatric NAFLD [76]. Despite the importance of secondary bile acids, little is known about their presence and the possible roles played in humans during infancy and childhood, warranting further studies.

Strengths of the current study include using both metagenomics and metabolomics for assessing the impact of the delivery mode and early nutrition on infant microbiota, and the clear documentation of when the infant formula was introduced to MF infants. However, the present study has some limitations that should be considered. For example, the participants were selected from a single cohort in the US and sampled at a single time point. Therefore, our findings may not represent the population elsewhere or at different time points. Additionally, while attempts were made to record the proportion of the diet made up of either human milk or formula, we could not obtain these data from all MF infants. Therefore, only categorical data were used to group by feeding practice. It is possible that infants who received only a small fraction of formula in the first several weeks of life were able to recover a microbiome that was closer to that of a BF infant, warranting further studies.

In conclusion, this study demonstrated that both delivery mode and feeding practice affected the infant’s fecal microbiome and metabolome at 6 weeks of age. Fecal microbiome alpha and beta diversity, taxonomic composition, and metabolic function of BF differed from that of infants fed all (FF) or some formula (MF). The microbiome and metabolomic profiles of MF infants were closer to those of FF than BF infants. Delivery mode influenced overall microbial community structure, taxonomic composition, individual fecal metabolites, and their represented metabolic pathways.

## 5. Future Studies

Larger longitudinal multi-omics studies are needed to determine the temporal dynamics of the effects of feeding practice and delivery mode on both microbiota composition and their potential short and long-term implications on infant and child health.

## Figures and Tables

**Table 1:** Demographics of study participants.

	BF		MF		FF			*p* Value	
	VD	CS	VD	CS	VD	CS	Feeding	Delivery	Interaction
n =	13	12	13	12	13	12	-	-	-
Male, n (%)	6 (46.2)	6 (50)	5 (38.5)	7 (58.3)	6 (46.2)	6 (50)	1	0.573	-
Gestational age at birth, wk	39.5 ± 0.39	39.8 ± 0.42	39.5 ± 0.39	39.6 ± 0.29	39.3 ± 0.29	39.2 ± 0.41	0.520	0.696	0.783
Weight-for-length z-score									
At birth	−0.12 ± 0.42	0.13 ± 0.54	−0.90 ± 0.34	−0.67 ± 0.47	−0.64 ± 0.33	−0.98 ± 0.39	0.091	0.898	0.732
At 6 wk	0.76 ± 0.33	0.08 ± 0.36	−0.18 ± 0.18	0.26 ± 0.40	0.03 ± 0.23	−0.03 ± 0.41	0.378	0.713	0.247
Antibiotic intake 2 wk prior to sample collection, n	1	0	0	0	0	2	0.346	0.951	-
Infant ethnicity, n							0.860	0.170	-
Non-Hispanic/Latino white	11	8	10	6	7	7			
Non-Hispanic/Latino non-white	1	3	0	5	4	3			
Hispanic/Latino	1	0	1	1	2	1			
Not reported	0	1	2	0	0	1			
Breastmilk consumed at 6 wk, %	100	100	46.4 ± 8.86 ^#^	61.6 ± 9.99 ^#^	0	0	-	-	-
Maternal age, year	32.5 ± 0.96	32.7 ± 1.33	30.7 ± 1.05	29.9 ± 1.63	28.2 ± 1.53	27.0 ± 1.87	0.003	0.590	0.888
Prepregnancy BMI	27.0 ± 1.50	29.5 ± 1.98	28.6 ± 2.22	31.6 ± 3.54	27.8 ± 2.16	35.8 ± 2.74	0.329	0.022	0.434

Data are presented as means ± SEMs, unless otherwise noted; ^#^ MF-VD, n = 11; MF-CS, n = 7. Abbreviations: BF, breastfed; CS, cesarean section; FF, formula-fed, MF, mixed-fed; VD, vaginally delivered.

**Table 2:** Diversity indices of fecal microbiota of exclusively breastfed, mixed-fed, and exclusively formula-fed infants at 6 weeks of age.

	BF		MF		FF			*p* Value	
	VD	CS	VD	CS	VD	CS	Feeding	Delivery	Interaction
Genus									
Shannon	0.75 ± 0.11	0.67 ± 0.16	1.33 ± 0.16	1.51 ± 0.12	1.35 ± 0.13	1.47 ± 0.13	<0.001	0.534	0.610
Inverse Simpson	1.85 ± 0.17	1.81 ± 0.31	3.13 ± 0.42	3.55 ± 0.45	3.04 ± 0.42	3.36 ± 0.71	<0.001	0.619	0.619
Evenness	0.31 ± 0.04	0.25 ± 0.06	0.43 ± 0.05	0.51 ± 0.03	0.42 ± 0.04	0.47 ± 0.03	<0.001	0.617	0.219
Species									
Shannon	1.11 ± 0.11	0.96 ± 0.12	1.67 ± 0.17	1.89 ± 0.18	1.77 ± 0.15	2.00 ± 0.11	<0.001	0.411	0.330
Inverse Simpson	2.56 ± 0.29	2.18 ± 0.27	4.03 ± 0.52	5.51 ± 1.00	4.70 ± 0.73	5.24 ± 0.70	<0.001	0.428	0.334
Evenness	0.40 ± 0.04	0.32 ± 0.03	0.45 ± 0.04	0.53 ± 0.03	0.47 ± 0.04	0.54 ± 0.02	<0.001	0.533	0.076

Data presented as mean ± SEM. Abbreviations: BF, breastfed; CS, cesarean section; FF, formula-fed, MF, mixed-fed; VD, vaginally delivered.

**Table 3:** Mode of feeding affected the potential metabolic pathways in the fecal microbiome of exclusively breastfed, mixed-fed, and exclusively formula-fed infants at 6 weeks of age.

	Total	FF vs. BF	MF vs. BF	MF vs. FF
Numbers of pathways that differed (*p* <0.05, *q* < 0.10)	155	117	112	8
MetaCyc superclass ^#^				
Biosynthesis	92	70	71	5
Biosynthesis/detoxification	3	2	2	0
Degradation/utilization/assimilation (DUA)	32	22	22	0
Generation of precursor metabolites and energy (GPME)	17	15	10	1
DUA/GPME	5	3	2	1
Others	6	5	5	1

^#^ 8 pathways were associated with >1 MetaCyc superclasses, including 3 belonged to both biosynthesis and detoxification, and 5 to both DUA and GPME. Abbreviations: BF, breastfed; FF, formula-fed, MF, mixed-fed.

**Table 4:** Mode of feeding affected the fecal metabolites in exclusively breastfed, mixed-fed, and exclusively formula-fed infants at 6 weeks of age.

	Total	FF vs. BF	MF vs. BF	MF vs. FF
Numbers of metabolites that differed				
(*p* < 0.05, *q* < 0.10)	577	543 (386|157)	517 (307|210)	3 (0|3)
Super-pathway				
Amino acid	97	91 (72|19)	86 (68|18)	0
Peptide	15	14 (12|2)	12 (11|1)	0
Carbohydrate	21	20 (7|13)	20 (8|12)	0
Energy	5	5 (3|2)	3 (2|1)	0
Lipid	231	222 (144|78)	206 (142|64)	2 (0|2)
Nucleotide	21	20 (17|3)	18 (16|2)	0
Cofactors and vitamins	34	30 (23|7)	33 (27|6)	0
Xenobiotics	47	41 (26|15)	42 (29|13)	1 (0|1)
Unnamed	106	100 (82|18)	97 (82|15)	0

Orange values indicate higher numbers of metabolites were higher in numerator group at *p* < 0.05, *q* < 0.10. Blue values indicate higher numbers of metabolites were lower in numerator group at *p* < 0.05, *q* < 0.10. Abbreviations: BF, breastfed; FF, formula-fed, MF, mixed-fed.

**Table 5:** Feeding mode affected di- and oligosaccharides in feces of exclusively breastfed, mixed-fed, and exclusively formula-fed infants at 6 weeks of age.

			Log_2_ Fold-Change		
Biochemical Name	Super-Pathway	Sub-Pathway	FF/BF	MF/BF	FF/MF
lactose	Carbohydrate	Disaccharides and Oligosaccharides	−2.94	−1.47	−1.43
lacto-N-fucopentaose II	Carbohydrate	Disaccharides and Oligosaccharides	−7.82	−5.64	−2.18
lacto-N-fucopentaose V	Carbohydrate	Disaccharides and Oligosaccharides	−3.47	−2.18	−1.29
lacto-N-tetraose	Carbohydrate	Disaccharides and Oligosaccharides	−4.64	−3.64	−0.92
3-sialyllactose	Carbohydrate	Disaccharides and Oligosaccharides	−3.06	−3.06	0.03
6′-sialyllactose	Carbohydrate	Disaccharides and Oligosaccharides	−4.64	-4.32	−0.56
2-fucosyllactose	Carbohydrate	Disaccharides and Oligosaccharides	−5.06	−4.06	−1.12
3-fucosyllactose	Carbohydrate	Disaccharides and Oligosaccharides	−9.20	−6.64	−2.56
sucrose	Carbohydrate	Disaccharides and Oligosaccharides	2.68	2.86	−0.18

Orange color indicates higher in FF or MF and blue indicates lower in FF or MF infants at *p* < 0.05 and *q* < 0.10. Abbreviations: BF, Breastfed; FF, formula-fed; MF, mixed-fed.

**Table 6:** Feeding mode affected fatty acids in feces of exclusively breastfed, mixed-fed, and exclusively formula-fed infants at 6 weeks of age.

			Log_2_ Fold-Change		
Biochemical Name	Super-Pathway	Sub-Pathway	FF/BF	MF/BF	FF/MF
valerate (5:0)	Lipid	Short Chain Fatty Acid	4.37	3.50	0.87
pentoic acid	Lipid	Short Chain Fatty Acid	1.10	1.23	−0.12
caproate (6:0)	Lipid	Medium Chain Fatty Acid	2.86	2.42	0.43
heptanoate (7:0)	Lipid	Medium Chain Fatty Acid	1.08	1.88	−0.79
caprylate (8:0)	Lipid	Medium Chain Fatty Acid	0.97	0.96	0.01
caprate (10:0)	Lipid	Medium Chain Fatty Acid	1.77	1.35	0.42
laurate (12:0)	Lipid	Medium Chain Fatty Acid	1.68	1.04	0.64
5-dodecenoate (12:1n7)	Lipid	Medium Chain Fatty Acid	−1.36	−1.40	0.04
myristate (14:0)	Lipid	LC-SFA	−1.09	−0.86	−0.22
pentadecanoate (15:0)	Lipid	LC-SFA	−2.40	−1.60	−0.81
palmitate (16:0)	Lipid	LC-SFA	−0.89	−0.76	−0.12
margarate (17:0)	Lipid	LC-SFA	−3.18	−2.47	−0.64
stearate (18:0)	Lipid	LC-SFA	−2.12	−1.74	−0.42
nonadecanoate (19:0)	Lipid	LC-SFA	−2.64	−2.00	−0.62
arachidate (20:0)	Lipid	LC-SFA	−1.25	−0.94	−0.34
myristoleate (14:1n5)	Lipid	LC-MUFA	−1.79	−1.60	−0.18
palmitoleate (16:1n7)	Lipid	LC-MUFA	−3.47	−2.84	−0.74
10-heptadecenoate (17:1n7)	Lipid	LC-MUFA	−3.06	−2.18	−0.89
oleate/vaccenate (18:1)	Lipid	LC-MUFA	−2.00	−1.51	−0.47
10-nonadecenoate (19:1n9)	Lipid	LC-MUFA	−3.84	−3.06	−0.71
eicosenoate (20:1)	Lipid	LC-MUFA	−3.47	−2.74	−0.74
erucate (22:1n9)	Lipid	LC-MUFA	−5.06	−3.84	−1.43
nervonate (24:1n9)	Lipid	LC-MUFA	−5.06	−3.64	−1.56
stearidonate (18:4n3)	Lipid	LC-PUFA (n3 and n6)	−3.18	−2.56	−0.62
eicosapentaenoate (EPA; 20:5n3)	Lipid	LC-PUFA (n3 and n6)	−4.06	−3.32	−0.79
heneicosapentaenoate (21:5n3)	Lipid	LC-PUFA (n3 and n6)	−1.25	−1.06	−0.18
docosapentaenoate (n3 DPA; 22:5n3)	Lipid	LC-PUFA (n3 and n6)	−2.94	−2.94	0.00
docosahexaenoate (DHA; 22:6n3)	Lipid	LC-PUFA (n3 and n6)	−2.64	−2.64	−0.04
docosatrienoate (22:3n3)	Lipid	LC-PUFA (n3 and n6)	−5.06	−4.06	−1.25
hexadecadienoate (16:2n6)	Lipid	LC-PUFA (n3 and n6)	−2.06	−1.64	−0.45
linoleate (18:2n6)	Lipid	LC-PUFA (n3 and n6)	−2.18	−1.69	−0.49
linolenate [alpha or gamma; (18:3n3 or 6)]	Lipid	LC-PUFA (n3 and n6)	−1.60	−1.36	−0.27
dihomo-linoleate (20:2n6)	Lipid	LC-PUFA (n3 and n6)	−4.64	−3.64	−0.97
dihomo-linolenate (20:3n3 or n6)	Lipid	LC-PUFA (n3 and n6)	−2.84	−2.84	0.04
arachidonate (20:4n6)	Lipid	LC-PUFA (n3 and n6)	−2.84	−2.74	−0.12
adrenate (22:4n6)	Lipid	LC-PUFA (n3 and n6)	−2.47	−2.12	−0.36
docosapentaenoate (n6 DPA; 22:5n6)	Lipid	LC-PUFA (n3 and n6)	−4.32	−3.64	−0.81
docosadienoate (22:2n6)	Lipid	LC-PUFA (n3 and n6)	−6.64	−4.64	−2.84
mead acid (20:3n9)	Lipid	LC-PUFA (n3 and n6)	1.85	2.47	−0.62

Orange color indicates higher in numerator group and blue indicates lower in numerator group at *p* < 0.05 and *q* < 0.10. Abbreviation: BF, breastfed; FF, formula-fed; MF; mixed-fed; LC-MUFA, long chain monounsaturated fatty acid; LC-PUFA, long chain polyunsaturated fatty acid; LC-SFA, long chain saturated fatty acid.

**Table 7:** Feeding mode affected metabolites derived from aromatic amino acids and lysine in feces of exclusively breastfed, mixed-fed, and exclusively formula-fed infants at 6 weeks of age.

			Log_2_ Fold-Change		
Biochemical Name	Super-Pathway	Sub-Pathway	FF/BF	MF/BF	FF/MF
3-methylhistidine	Amino Acid	Histidine metabolism	−1.69	−1.40	−0.29
N-acetyl-1-methylhistidine	Amino Acid	Histidine metabolism	0.73	1.14	−0.42
hydantoin-5-propionate	Amino Acid	Histidine metabolism	1.52	1.55	−0.03
imidazole propionate	Amino Acid	Histidine metabolismb	2.82	1.50	1.32
formiminoglutamate	Amino Acid	Histidine metabolism	4.41	4.28	0.14
carnosine	Amino Acid	Histidine metabolism	−1.74	−1.40	−0.36
histamine	Amino Acid	Histidine metabolism	−0.56	-2.32	1.73
4-imidazoleacetate	Amino Acid	Histidine metabolism	2.76	2.11	0.65
N-acetylhistamine	Amino Acid	Histidine metabolism	1.56	0.10	1.46
lysine	Amino Acid	Lysine metabolism	1.95	1.23	0.72
N2-acetyllysine	Amino Acid	Lysine metabolism	3.20	2.28	0.93
N6-acetyllysine	Amino Acid	Lysine metabolism	1.01	1.08	−0.06
N6-formyllysine	Amino Acid	Lysine metabolism	3.96	3.38	0.58
N6-carboxyethyllysine	Amino Acid	Lysine metabolism	2.65	2.30	0.34
hydroxy-N6,N6,N6-trimethyllysine	Amino Acid	Lysine metabolism	−2.64	−1.94	−0.71
fructosyllysine	Amino Acid	Lysine metabolism	5.45	5.27	0.18
saccharopine	Amino Acid	Lysine metabolism	2.42	2.11	0.30
2-aminoadipate	Amino Acid	Lysine metabolism	0.20	1.07	−0.86
pipecolate	Amino Acid	Lysine metabolism	4.49	3.58	0.91
6-oxopiperidine-2-carboxylate	Amino Acid	Lysine metabolism	1.54	1.81	−0.27
cadaverine	Amino Acid	Lysine metabolism	2.31	2.08	0.24
N-acetyl-cadaverine	Amino Acid	Lysine metabolism	2.84	2.38	0.46
5-aminovalerate	Amino Acid	Lysine metabolism	5.45	3.78	1.67
N,N,N-trimethyl-5-aminovalerate	Amino Acid	Lysine metabolism	3.75	3.33	0.42
phenylacetate	Amino Acid	Phenylalanine metabolism	5.10	4.17	0.93
4-hydroxyphenylacetate	Amino Acid	Phenylalanine metabolism	4.18	3.26	0.92
4-hydroxyphenylpyruvate	Amino Acid	Tyrosine metabolism	−0.64	−1.00	0.34
3-(4-hydroxyphenyl)lactate	Amino Acid	Tyrosine metabolism	−1.60	−1.36	−0.25
o-Tyrosine	Amino Acid	Tyrosine metabolism	1.03	1.09	−0.06
dopamine 3-O-sulfate	Amino Acid	Tyrosine metabolism	0.66	1.08	−0.42
tyramine O-sulfate	Amino Acid	Tyrosine metabolism	1.72	1.58	0.14
N-formylphenylalanine	Amino Acid	Tyrosine metabolism	0.72	0.86	−0.14
C-glycosyltryptophan	Amino Acid	Tryptophan metabolism	−2.12	−1.60	−0.51
tryptophan betaine	Amino Acid	Tryptophan metabolism	−4.32	−2.12	−2.18
N-formylanthranilic acid	Amino Acid	Tryptophan metabolism	1.77	1.05	0.72
xanthurenate	Amino Acid	Tryptophan metabolism	−1.03	−0.76	−0.29
picolinate	Amino Acid	Tryptophan metabolism	2.10	1.98	0.12
tryptamine	Amino Acid	Tryptophan metabolism	3.83	1.83	2.00
indoleacetate	Amino Acid	Tryptophan metabolism	3.10	2.84	0.26
indole-3-carboxylate	Amino Acid	Tryptophan metabolism	1.33	1.00	0.33

Orange color indicates higher in FF or MF and blue indicates lower in FF or MF infants at *p* < 0.05 and *q* < 0.10. Abbreviations: BF, Breastfed; FF, formula-fed; MF, mixed-fed.

**Table 8:** Feeding mode affected bile acid metabolites in feces of exclusively breastfed, mixed-fed, and exclusively formula-fed infants at 6 weeks of age.

			Log_2_ Fold-Change		
Biochemical Name	Super-Pathway	Sub-Pathway	FF/BF	MF/BF	FF/MF
taurocholate	Lipid	Primary Bile Acid Metabolism	−2.47	−2.32	−0.10
taurochenodeoxycholate	Lipid	Primary Bile Acid Metabolism	−2.47	−2.12	−0.38
ursodeoxycholate	Lipid	Secondary Bile Acid Metabolism	2.88	1.46	1.42
isoursodeoxycholate	Lipid	Secondary Bile Acid Metabolism	2.03	0.72	1.31
tauroursodeoxycholic acid sulfate	Lipid	Secondary Bile Acid Metabolism	1.09	0.38	0.71
7,12-diketolithocholate	Lipid	Secondary Bile Acid Metabolism	1.75	0.03	1.72
7-ketolithocholate	Lipid	Secondary Bile Acid Metabolism	1.76	−0.25	2.00
hyocholate	Lipid	Secondary Bile Acid Metabolism	1.23	0.59	0.63
3-dehydrocholate	Lipid	Secondary Bile Acid Metabolism	1.37	0.55	0.82
taurocholenate sulfate	Lipid	Secondary Bile Acid Metabolism	−2.18	−2.40	0.25
7-ketodeoxycholate	Lipid	Secondary Bile Acid Metabolism	2.18	0.52	1.67
ursocholate	Lipid	Secondary Bile Acid Metabolism	3.00	1.12	1.88

Orange color indicates higher in FF or MF and blue indicates lower in FF or MF infants at *p* < 0.05 and *q* < 0.10. Abbreviations: BF, Breastfed; FF, formula-fed; MF, mixed-fed.

**Table 9:** Enriched metabolic pathways associated with feeding mode from host, microbiota or co-metabolism in the feces of exclusively breastfed, mixed-fed, and exclusively formula-fed infants at 6 weeks of age.

		FF vs. BF		MF vs. BF			
		Match		Match			
ID	KEGG Pathway	Status	*p* Value	Status	*p* Value	Class	Origin
hsa00592	alpha-Linolenic acid metabolism	1 in 13	0.016	1 in 13	0.016	Lipid metabolism	Host
ko00130	Ubiquinone/other terpenoid-quinone biosyn.	5 in 59	<0.001	5 in 59	<0.001	Metabolism of cofactors and vitamins	Microbiota
ko00290	Valine, leucine, and isoleucine biosynthesis	3 in 23	0.002	2 in 23	0.021	Amino acid metabolism	Microbiota
ko00340	Histidine metabolism	3 in 32	0.004	2 in 32	0.039	Amino acid metabolism	Microbiota
ko00310	Lysine degradation	3 in 52	0.016	3 in 52	0.014	Amino acid metabolism	Microbiota
ko00040	Pentose and glucuronate interconversions	3 in 56	0.02	3 in 56	0.017	Carbohydrate metabolism	Microbiota
ko01040	Biosynthesis of unsaturated fatty acids	10 in 36	<0.001	10 in 36	<0.001	Lipid metabolism	Co-metab
ko00232	Caffeine metabolism	7 in 15	<0.001	6 in 15	<0.001	Biosynthesis of other secondary metabolites	Co-metab
ko00250	Alanine, aspartate and glutamate metabolism	8 in 28	<0.001	7 in 28	<0.001	Amino acid metabolism	Co-metab
ko00240	Pyrimidine metabolism	11 in 61	<0.001	10 in 61	<0.001	Nucleotide metabolism	Co-metab
ko00230	Purine metabolism	12 in 86	<0.001	9 in 86	<0.001	Nucleotide metabolism	Co-metab
ko00430	Taurine and hypotaurine metabolism	6 in 22	<0.001	6 in 22	<0.001	Biosynthesis of other secondary metabolites	Co-metab
ko00330	Arginine and proline metabolism	9 in 63	<0.001	8 in 63	<0.001	Amino acid metabolism	Co-metab
ko00470	D-Amino acid metabolism	8 in 56	<0.001	8 in 56	<0.001	Metab of other amino acids	Co-metab
ko00270	Cysteine and methionine metabolism	8 in 58	<0.001	8 in 58	<0.001	Amino acid metabolism	Co-metab
ko00260	Glycine, serine, and threonine metabolism	7 in 47	<0.001	7 in 47	<0.001	Amino acid metabolism	Co-metab
ko00600	Sphingolipid metabolism	5 in 23	<0.001	6 in 23	<0.001	Lipid metabolism	Co-metab
ko00410	beta-Alanine metabolism	5 in 25	<0.001	5 in 25	<0.001	Metabolism of other amino acids	Co-metab
ko00310	Lysine degradation	7 in 52	<0.001	7 in 52	<0.001	Amino acid metabolism	Co-metab
ko00970	Aminoacyl-tRNA biosynthesis	7 in 52	<0.001	7 in 52	<0.001	Translation	Co-metab
ko00770	Pantothenate and CoA biosynthesis	5 in 27	<0.001	5 in 27	<0.001	Metabolism of cofactors and vitamins	Co-metab
ko00340	Histidine metabolism	5 in 33	0.003	6 in 33	<0.001	Amino acid metabolism	Co-metab
ko00591	Linoleic acid metabolism	3 in 10	0.003	3 in 10	0.002	Lipid metabolism	Co-metab
ko00564	Glycerophospholipid metabolism	6 in 50	0.003	6 in 50	0.003	Lipid metabolism	Co-metab
ko00630	Glyoxylate and dicarboxylate metabolism	6 in 56	0.006	5 in 53	0.018	Carbohydrate metabolism	Co-metab
ko00480	Glutathione metabolism	4 in 32	0.014	4 in 32	0.013	Metabolism of other amino acids	Co-metab
ko00061	Fatty acid biosynthesis	5 in 53	0.019	5 in 53	0.018	Lipid metabolism	Co-metab
ko00660	C5-Branched dibasic acid metabolism	3 in 22	0.026	3 in 22	0.025	Carbohydrate metabolism	Co-metab
ko00220	Arginine biosynthesis	3 in 23	0.029	4 in 23	0.004	Amino acid metabolism	Co-metab
ko00052	Galactose metabolismb	4 in 46	0.046	4 in 46	0.043	Carbohydrate metabolism	Co-metab
ko00920	Sulfur metabolism	4 in 30	0.011	2 in 30	0.216	Energy metabolism	Co-metab
ko00100	Steroid biosynthesis	5 in 49	0.014	4 in 49	0.052	Lipid metabolism	Co-metab
ko00020	Citrate cycle (TCA cycle)	3 in 20	0.02	2 in 20	0.113	Carbohydrate metabolism	Co-metab
ko00290	Valine, leucine and isoleucine biosynthesis	3 in 23	0.029	2 in 23	0.142	Amino acid metabolism	Co-metab
ko00750	Vitamin B6 metabolism	2 in 23	0.147	3 in 23	0.028	Metabolism of cofactors and vitamins	Co-metab
ko00730	Thiamine metabolism	3 in 29	0.053	3 in 29	0.05	Metabolism of cofactors and vitamins	Co-metab
ko00261	Monobactam biosynthesis	3 in 29	0.053	3 in 29	0.05	Biosynthesis of other secondary metabolites	Co-metab

Abbreviations: BF, Breastfed; Co-metab, co-metabolism; FF, formula-fed; MF, mixed-fed.

**Table 10:** Delivery mode affected fecal metabolites of infants at 6 weeks of age independent of feeding mode.

Biochemical Name	Super-Pathway	Sub-Pathway	Log_2_ Fold-Change CS/VD
alpha-ketoglutaramate	Amino acid	Glutamate metabolism	−1.50
S-1-pyrroline-5-carboxylate	Amino acid	Glutamate metabolism	−1.28
formiminoglutamate	Amino acid	Histidine metabolism	−1.85
mannose	Carbohydrate	Fructose, mannose, and galactosemetabolism	−1.17
fuculose	Carbohydrate	Fructose, mannose, and galactose metabolism	−1.34
fucose	Carbohydrate	Aminosugar metabolism	−1.43
N-acetylglucosamine/N-acetylgalactosamine	Carbohydrate	Aminosugar metabolism	−1.05
(12 or 13)-methylmyristate (a15:0 or i15:0)	Lipid	Fatty acid, branched	−2.23
3-hydroxypalmitate	Lipid	Fatty acid, monohydroxy	−1.50
2S,3R-dihydroxybutyrate	Lipid	Fatty acid, dihydroxy	−1.11
chiro-inositol	Lipid	Inositol metabolism	1.04
glycerophosphoserine	Lipid	Phospholipid metabolism	−1.22
trimethylamine N-oxide	Lipid	Phospholipid metabolism	−1.36
1-pentadecanoylglycerol (15:0)	Lipid	Monoacylglycerol	−1.43
3-ketosphinganine	Lipid	Sphingolipid synthesis	−2.45
taurolithocholate 3-sulfate	Lipid	Secondary bile acidmetabolism	1.90
taurochenodeoxycholic acid 3-sulfate	Lipid	Secondary bile acid metabolism	2.63
2′-deoxyinosine	Nucleotide	Purine metabolism, (hypo)xanthine/inosine containing	−1.34
1-methyladenine	Nucleotide	Purine metabolism, adenine containing	−1.05
2′-deoxyguanosine	Nucleotide	Purine metabolism, guanine containing	−1.20
uridine	Nucleotide	Pyrimidine metabolism, uracil containing	−1.06
2′-deoxyuridine	Nucleotide	Pyrimidine metabolism, uracil containing	−1.11
thymidine	Nucleotide	Pyrimidine metabolism, thymine containing	−1.03
pantoate	Cofactors and vitamins	Pantothenate and coenzyme A metabolism	−1.69
2-isopropylmalate	Xenobiotics	Food component/plant	−1.11
histidinol	Xenobiotics	Food component/lant	−1.68
tartarate	Xenobiotics	Food component/plant	−1.18
glutamyl-meso-diaminopimelate	Xenobiotics	Bacterial/fungal	−1.34
N-propionylmethionine	Xenobiotics	Chemical	−1.23
X-23734	N/A	N/A	−2.10
X-24660	N/A	N/A	1.29
X-24669	N/A	N/A	2.08
X-25185	N/A	N/A	−2.19
X-25436	N/A	N/A	1.12
X-25491	N/A	N/A	−1.50

Orange color indicates higher in CS and blue indicates lower in CS at *p* < 0.05 and *q* < 0.10. Only the metabolites with |log_2_ fold-change| > 1 were shown. Abbreviations: CS, cesarean section; VD, vaginally delivered.

**Table 11:** Enriched metabolic pathways associated with delivery mode from microbiota or co-metabolism in feces at infants at 6 weeks of age.

ID	KEGG Pathway	Match Status	*p* Value	Class	Origin
ko00290	Valine, leucine and isoleucine biosynthesis	1 in 23	0.041	Amino acid metabolism	Microbiota
ko00770	Pantothenate and CoA biosynthesis	1 in 27	0.048	metabolism of cofactors and vitamins	Microbiota
ko00620	Pyruvate metabolism	1 in 28	0.049	Carbohydrate metabolism	Microbiota
ko00600	Sphingolipid metabolism	3 in 23	<0.001	Lipid metabolism	Co-metabolism
ko00240	Pyrimidine metabolism	3 in 61	0.002	Nucleotide metabolism	Co-metabolism
ko00250	Alanine, aspartate and glutamate metabolism	2 in 28	0.005	Amino acid metabolism	Co-metabolism
ko00230	Purine metabolism	2 in 86	0.043	Nucleotide metabolism	Co-metabolism

Abbreviations: CoA, coenzyme A.

## Data Availability

The raw microbiome sequencing reads used in the manuscript are available at NCBI Sequence Read Archive under the BioProject accession number PRJNA778397. Metabolome data and sample metadata are provided in Supplementary Tables S5 and S6.

## Supplementary Materials

The following support information can be downloaded at: https://www.mdpi.com/article/10.3390/microorganisms13010166/s1, Table S1: Relative abundances of microbial genera in feces of exclusively breastfed, mixed-fed, and exclusively formula-fed infants at 6 weeks of age; Table S2: relative abundances of microbial species in feces of exclusively breastfed, mixed-fed, and exclusively formula-fed infants at 6 weeks of age; Table S3: Metabolites affected by feeding mode in feces of exclusively breastfed, mixed-fed, and exclusively formula-fed infants at 6 weeks of age; Table S4: Infant fecal metabolites affected by delivery mode at 6 weeks of age; Table S5: peak identities and areas under the curve of fecal metabolites of exclusively breastfed, mixed-fed, and exclusively formula-fed infants at 6 weeks of age; Table S6: Sample metadata; Figure S1: Fecal metabolic pathways differed between BF and FF infants with MF being intermediate at 6 weeks of age; Figure S2: Fecal metabolic pathways differed between BF and MF infants with FF being intermediate at 6 weeks of age.
