# Supplementary material for: Fecal Microbiome and Metabolomic Profiles of Mixed-Fed Infants Are More Similar to Formula-Fed than Breastfed Infants
Source: Microorganisms. 2025 Jan 14;13(1):166. doi: 10.3390/microorganisms13010166 (PMC11767595; doi:10.3390/microorganisms13010166)
Supplement: Supplementary file 1 [file microorganisms-13-00166-s001.zip › Supplementary Figures S1 and S2.pdf]

Supplementary Figures

Mei Wang, Negin Valizadegan, Christopher J. Fields, Sharon M. Donovan

Fecal Microbiome and Metabolomic Profiles of Mixed-fed Infants are More Similar to Formula-fed than Breastfed Infants

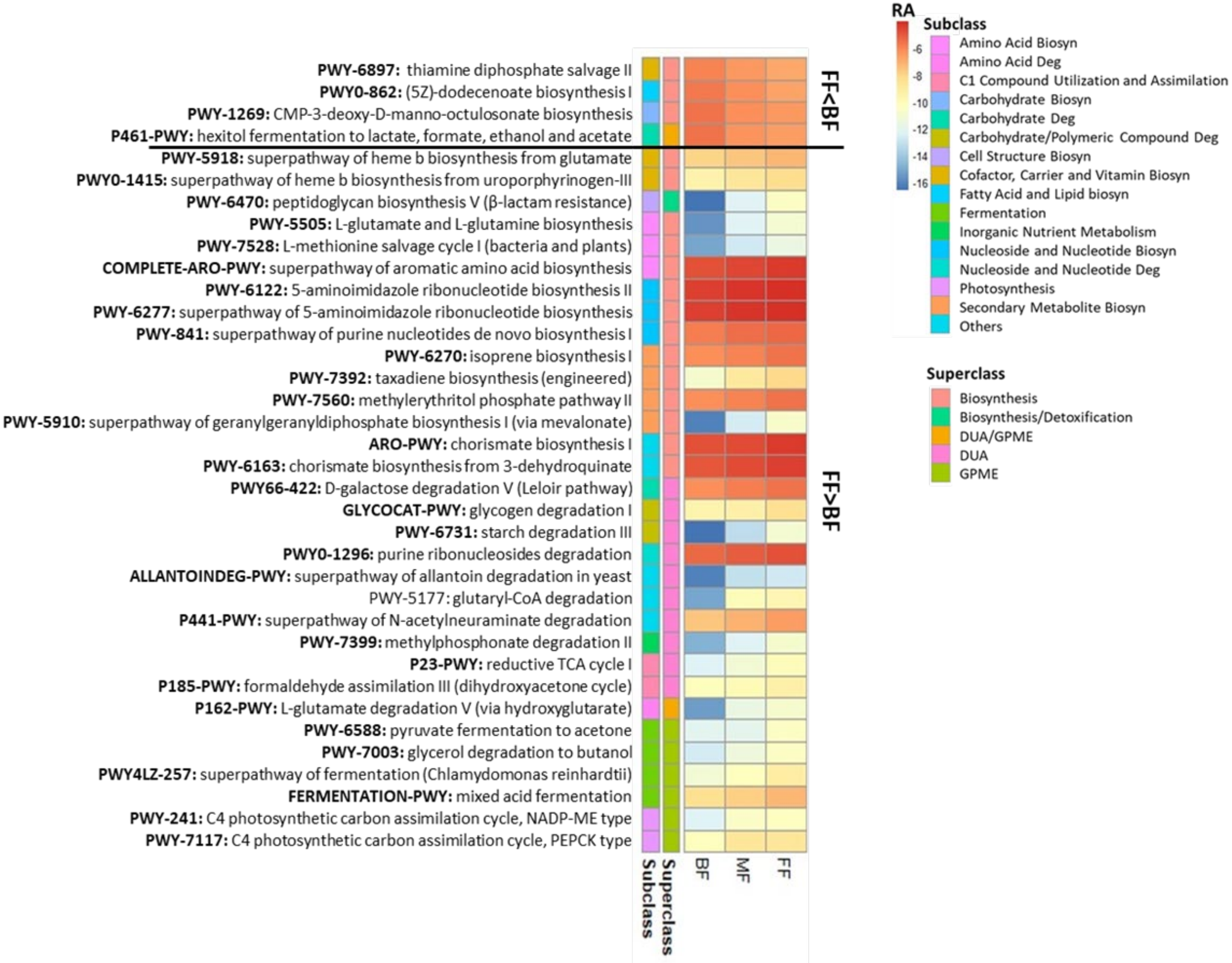

Figure S1. Fecal metabolic pathways differed between BF and FF Infants with MF being intermediate at 6-weeks-of age ($p < 0.05$, $q < 0.10$). Biosyn, biosynthesis; BF, breastfed; Deg, degradation; DUA, degradation/utilization/assimilation; FF, formula-fed; GPME, generation of precursor

Supplementary Figures

Mei Wang, Negin Valizadegan, Christopher J. Fields, Sharon M. Donovan

Fecal Microbiome and Metabolomic Profiles of Mixed-fed Infants are More Similar to Formula-fed than Breastfed Infants

metabolites and energy; RA, log_2 (mean relative abundance)

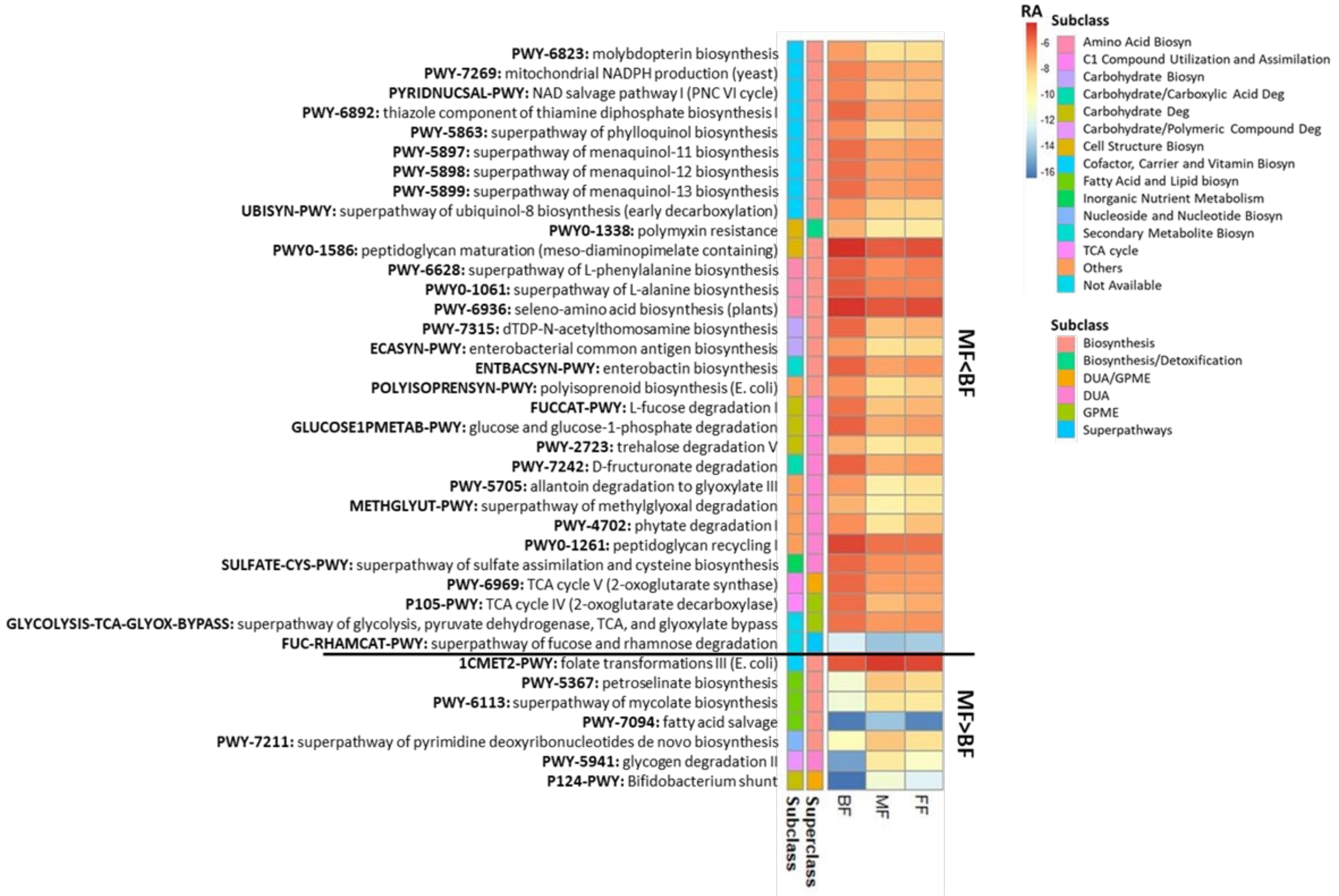

Supplementary Figures
Mei Wang, Negin Valizadegan, Christopher J. Fields, Sharon M. Donovan
Fecal Microbiome and Metabolomic Profiles of Mixed-fed Infants are More Similar to Formula-fed than Breastfed Infants

Figure S2. Fecal metabolic pathways differed between BF and MF infants with FF being intermediate at 6-weeks- of age ($p < 0.05$, $q < 0.10$). Biosyn, biosynthesis; BF, breastfed; Deg, degradation; DUA, degradation/utilization/assimilation; GPME, generation of precursor metabolites and energy; MF, mixed-fed; RA, log2 (mean relative abundance).
